# Genetic and clinical study of myeloperoxidase's association with coronary artery disease

**DOI:** 10.1186/s43044-024-00457-7

**Published:** 2024-02-21

**Authors:** Lina N. Adam, Omar A. M. Al-Habib, Ashur Y. Oraha, Mudhir S. Shekha

**Affiliations:** 1 Department of Biology, College of Science, University of Zakho, Duhok, Kurdistan Region Iraq; 2 Department of Biology, College of Science, University of Nawroz, Duhok, Kurdistan Region Iraq; 3 Department of Cardiothoracic and Vascular Surgery, College of Medicine, University of Duhok, Duhok, Kurdistan Region Iraq; 4 Department of Medical Cell Biology, Uppsala University, Uppsala, Sweden; 5 Department of Biology, College of Science, Salahaddin University-Erbil, Erbil, Kurdistan Region Iraq

**Keywords:** Myeloperoxidase (MPO), Coronary artery disease (CAD), Biomarkers, Cardiovascular risk, Genetic variation

## Abstract

**Background:**

Unraveling myeloperoxidase’s (MPO) correlation with coronary artery disease (CAD) and genetic variations, this study seeks to enhance diagnostic precision and therapeutic strategies.

**Results:**

CAD patients were found to be older and more male than controls. Several clinical parameters, including glucose, total bilirubin, alkaline phosphatase, creatinine, and troponin levels, showed significant variations. Moreover, CAD patients had lower red cell distribution width (RDW%) and mean platelet volume (MPV) than controls. Serum MPO levels did not differ significantly between CAD patients and controls, and no correlation was found with other clinical parameters except for glucose, creatinine, and total bilirubin.

**Conclusions:**

The data suggest that serum MPO levels are not substantially related to CAD patients, as indicated by lower MPO levels in CAD patients compared to controls. While highlighting the potential of MPV and RDW% as predictors of severe atherosclerosis in CAD. Further research is needed to validate the diagnostic and prognostic value of RDW%, MPV, and MPO levels in CAD.

***Trial registration***: 15092021-9-12. Registered 15 September 2021.

## Background

Cardiovascular diseases (CVDs) have been identified as the leading cause of mortality globally by the World Health Organization (WHO), Pérez et al. [1]. Coronary artery disease (CAD), primarily caused by atherosclerosis, occurs when plaque accumulates within the coronary arteries, leading to partial or complete blood flow restriction and subsequent reduction in oxygen and nutrient supply to the myocardium [2]. The complex cascade of events involved in developing atherosclerotic plaque, from early endothelial dysfunction to mature atheroma formation, rupture, and inflammatory processes, necessitates a comprehensive understanding of the underlying variables [3]. Such knowledge is essential for identifying precise cardiovascular risk predictors and potential therapeutic intervention targets [4].

Myeloperoxidase (MPO), an enzyme produced by leukocytes, plays a critical role in innate immunity by catalyzing the synthesis of oxidative reactants to combat infections [5]. Recent studies have also implicated MPO in the promotion and progression of atherosclerosis [6]; it has been shown to contribute to the instability of atherosclerotic plaques and impair endothelial function, thereby playing a pathogenic role in atherogenesis and contributing to oxidative stress [7, 8]. Furthermore, MPO can utilize nitric oxide as a physiological substrate, potentially leading to endothelial dysfunction [9]. Conversely, genetic variations impacting inflammatory biomarkers maintain stability over an individual's lifespan, are less susceptible to external environmental and medical influences, and are likely to have a lasting impact on prolonged exposure to inflammation.

Given MPO's emerging role as a potential biomarker and therapeutic target for CVD [10]. Therefore, this study aimed to investigate the relationship between serum MPO levels and coronary artery disease. It sought to assess clinical characteristics, biochemical parameters, and hematological markers associated with CAD and analyze the association between MPO levels and disease severity. Additionally, the study aimed to identify genetic variations in the MPO gene and evaluate their potential relevance to CAD. The findings aimed to enhance understanding of CAD pathophysiology and provide insights into diagnostic and prognostic markers for the disease.

## Methods

### Ethical approval and subject recruitment

The study protocol for this research was granted ethical approval by the Research Ethics Committee of the General Directorate of Health in Duhok, Iraq (Trial registration: 15092021-9-12. Registered 15 September 2021). Before their participation, all subjects were provided with comprehensive information regarding the study's objectives and the potential risks involved. Subsequently, each participant provided informed consent by signing a consent form.

### Study design and participant selection

This case–control research was conducted at the Cardiac Center, Azadi Teaching Hospital in Duhok, Iraq, from August to November 2021. A total of 70 patients who had confirmed CAD based on their coronary angiography reports were included in the patient group. CAD was determined if at least one of the main coronary arteries exhibited a 70% or higher luminal stenosis. For the control group, 30 volunteers matched in age and gender were recruited from individuals who had previously visited the cardiac center. These individuals underwent angiography, which revealed no luminal stenosis, thus confirming their normal coronary arteries. Moreover, Participants who meet any of the following conditions were excluded from the study: pregnant or lactating women, those unable to provide a sufficient blood sample for analysis, individuals with congenital heart disease, and those with incomplete medical records or unable to provide informed consent. These criteria were applied during the subject recruitment process to ensure the selection of an appropriate and eligible study population.

The substantial disparity in the number of CAD patients compared to control participants can be attributed to the high prevalence of CAD cases seeking medical attention, in contrast to the limited number of individuals without CAD who presented themselves for angiography as part of the diagnostic process for chest pain, and subsequently, were found to have normal angiographic results. Conducting angiography on individuals without CAD symptoms is not only operationally difficult but also causes discomfort and incurs substantial costs.

Notably, all participants, both cases and controls, were of Iraqi origin. Additionally, it is important to mention that most participants in both groups received medications for coronary artery disease, hypertension, and dyslipidemia, as appropriate for their respective conditions.

### Data collection and sample processing

A standardized questionnaire was administered to collect clinical information from the participants. The questionnaire included demographic and anthropometric data, risk factors for atherosclerosis, personal and family medical history, and details on current medication usage. Participants were specifically asked to report any medications taken, including the oral contraceptive pill and any medical treatments or COVID-19 vaccinations, received within the previous three months, as reported in our earlier study [11]. Approximately 10 mL of blood was collected from the all the subjects before the coronary angiography and/or surgery and stored in plain and EDTA vials. The Serum was separated by centrifugation at 1500 g for 5 min and stored at – 30 °C until further processing.

### Biochemical parameters

A comprehensive analysis of complete blood count and biochemical parameters was conducted to assess various health indicators, including lipid profile, renal function, cardiac function, kidney function, and serum glucose levels. These measurements were performed using a fully automated biochemical analyzer (Cobas 6000, HITACHI).

In addition, Serum total Myeloperoxidase (MPO) activity was determined using the enzyme-linked immunosorbent assay (ELISA) method. The Navand Salamat Myeloperoxidase (MPO) Activity Assay Kit (Nampox™), Iran, was utilized. The assay followed the manufacturer's instructions, utilizing an ELISA plate and reader capable of measuring absorbance at 650 nm wavelength.

## Reference range

The following reference ranges have been used in this study, Biochemical parameters: Renal function tests Urea > 50 mg/dl, creatinine > 1.2 mg/dl, liver function tests SGPT (ALT) > 41 U/L, SGOT (AST) > 40 U/L, Total Bilirubin > 1.2 mg/dl, ALP > 129 U/L, LDH > 225 U/L, Lipid profile Cholesterol > 200 mg/dl, Triglyceride > 200 mg/dl, HDL > 55 mg/dl, LDL 100 mg/dl, Cardiac function tests CRP > 5 mg/L, CKmB > 25 U/L, Troponin > 0.3 ng/mL, Glucose > 120 mg/dl. Hematological parameters: RBC 3.5–5.5 10^12^/l, WBC3.5–10 10^9^/l, GRAN1.2–8 10^9^/l, LYM 5–5.5 10^9^/l, MID 1–1.5 10^9^/l, HB 11.5–16.5 g/dl, HCT 35–55%, MCV 75–100 fl, MCH 25–35 pg, MCHC31-38 g/dl, RDW% 1–16%, RDWa 30–150 fl, PLT 100–400 10^9^/l, PCT 0.01–9.99%, MPV 8–11 fl, PDW 0.1–9.99 fl, PLCR 0.1–9.99%. [12].

### DNA isolation and polymerase chain reaction (PCR)

Out of the initial collection of 100 blood samples, a subset of 11 samples was deliberately employed for DNA isolation and subsequent Polymerase Chain Reaction (PCR) analysis. The decision to work with this reduced sample size was motivated by several considerations, each of which emphasizes the robustness and uniqueness of the study design. Genomic DNA was isolated from whole blood following the manufacturer's instructions, utilizing the Promega Wizard® Genomic DNA Purification Kit (Promega/USA). Polymerase chain reaction (PCR) was used to amplify target DNA fragments. All primers used were purchased from Integrated DNA Technologies, USA. Two primers were used, forward 5′-CGG TAT AGG CAC ACA ATG GTG AG-3′ and reverse 5′-GCA ATG GTT CAA GCG ATT CTT C-3′.

**Fig. 1:**
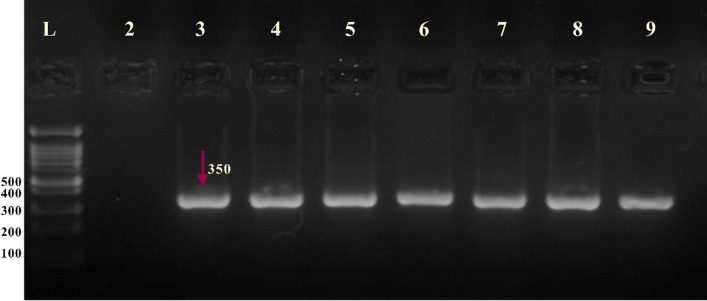
Myeloperoxidase (MPO)-463G/A polymorphism. Lane L: a standard 100-bp marker, Lanes 2: negative control, Lanes 3–9 MPO DNA fragments 350 bp

The genotypes were determined by visualizing the amplified DNA fragments on a 2% high-resolution agarose gel. The gel was stained with a safe gel stain dye (SYBR™) and examined under ultraviolet (UV) light. The length of the amplified DNA fragment was determined to be 350 bp (Fig. 1).

The PCR reaction was performed in a 25 μL reaction mixture containing; 12.5 μl master mix, 1.5 μl forward primer, 1.5 μl reverse primers, and 3 μl of isolated DNA. Finally, 6.5 μl of nuclease-free water was added to obtain 25 μL of total PCR mixture volume. PCR was carried out in Thermal Cycler (UK); the assay's PCR cycling conditions were 94 °C for 4 min as initial denaturation, followed by 35 cycles at 94 °C for 30 s, 56 °C for 35 s, and 72 °C for 1min, with a final extension step at 72 °C for 7 min. The length of the amplified DNA fragment was 350 bp and was verified by running 3 μl of PCR product on a 2% agarose gel (Fig. 1). The MPO SNP rs2333227 polymorphism was confirmed through Sanger sequencing of PCR products using the Genetic Analyzer 3500 (Macrogen/South Korea). This robust approach ensures accurate identification of genetic variants, such as single nucleotide polymorphisms (SNPs), and enhances the credibility of our findings.

### Data processing and statistical analysis

GraphPad Prism Statistics for Windows version 9.3.1 was used for statistical analysis. An independent sample t-test, Mann–Whitney test, and Spearman correlations were used to assess continuous data. The mean ± standard deviation (SD) of continuous variables with normal distributions were presented (SD). The Normality and Lognormality Tests used the Kolmogorov–Smirnov test to determine the normality of the variable distribution. Moreover, Receiver Operating Characteristic (ROC) curves were constructed to determine optimal cut-off values for each biomarker related to coronary artery disease (CAD) risk markers and the severity of CAD, using the highest Youden index. Statistically significant was defined as a two-tailed *P*-value < 0.05. Finally, Mutation Surveyor V5.1.2 analyzed the transcript variants at sequence length that a single nucleotide changed.

## Results

### Characteristics of the study population

The detailed baseline characteristics of the study had been published previously [11]. The clinical data of the study are summarized in Table 1. There was a statistically significant difference in age distribution between the two groups: CAD patients had a mean ± SD age of 58.8 ± 9.9 years, ranging from 41 to 85 years, while the control group had a mean ± SD age of 51.6 ± 10.8 years, ranging from 33 to 75 years (*P* = 0.0036). Biochemical parameters comparing patients with CAD and controls demonstrated several significant differences, as shown in Table 1. In CAD patients, Total Bilirubin, ALP (Alkaline Phosphatase), Glucose, Troponin, and creatinine levels were significantly higher than in controls (*P* = 0.009, *P* = 0.0405, *P* = 0.0042, *P* = 0.0042, and *P* = 0.0062, respectively). Additionally, Table 2 showed that the Mean Platelet Volume (MPV) and Red Cell Distribution Width (RDW%) were considerably greater in the control group compared to CAD patients (*P* < 0.05).

**Table 1:** Clinical characteristics of the studied population

Variables	CAD (*n* = 70)	Control (*n* = 30)	*P*-value
Age (Years)	58.8 ± 9.9	51.6 ± 10.8	0.0036*
Age range	41–85	33–75	–
*Renal function tests*			
urea (mg/dL)	36.6 ± 17.4	30.8 ± 10.1	0.1113
Creatinine (mg/dL)	1.9 ± 7.9	0.8 ± 0.3	0.0062*
*Liver function tests*			
GPT (U/L)	12.4 ± 8	9.7 ± 4.8	0.1500
GOT (U/L)	25.5 ± 22.9	20.4 ± 10	0.1823
Total Bilirubin (mg/dL)	0.5 ± 0.3	0.4 ± 0.2	0.0090*
Alp (U/L)	78.5 ± 21.9	72.5 ± 24	0.0405*
LDH (U/L)	235 ± 185.3	198.6 ± 124.9	0.4205
*Lipid profile and lipid ratios*			
Cholesterol (mg/dL)	154.5 ± 42.5	156.9 ± 37.9	0.8268
TG (mg/dL)	172.2 ± 98.6	190.5 ± 81.8	0.2648
HDL (mg/dL)	36.3 ± 7.8	38.1 ± 12.6	0.9806
LDL (mg/dL)	86.5 ± 38.7	80.3 ± 32.3	0.5282
TG/HDL-C ratio	5.3 ± 4.4	5.7 ± 3.5	0.3619
LDL-C/HDL-C ratio	2.5 ± 1.4	2.2 ± 0.9	0.6711
*Cardiac function tests*			
CRP (mg/L)	22.7 ± 65.3	6.5 ± 9.6	0.2001
CKmB (U/L)	25.1 ± 41.3	18.7 ± 25.3	0.2729
Troponin (ng/mL)	1.5 ± 4.7	0.1 ± 0.01	0.0042*
Glucose (mg/dL)	154 ± 77.6	110.1 ± 46.5	0.0042*

Results are expressed as numbers or means ± SD. *Statistical significance

**Table 2:** A comparison of hematological parameters between CAD and controls

Parameters	CAD (*n* = 70)	Control (*n* = 30)	*P*-value
RBC (10^12^/l)	5 ± 0.6	5.1 ± 0.63	0.6360
WBC (10^9^/l)	8.8 ± 2.8	8 ± 2.6	0.0806
GRAN (10^9^/l)	6 ± 2.3	6.9 ± 10.1	0.1166
LYM (10^9^/l)	2.4 ± 0.8	8.5 ± 14.1	0.9420
MID (10^9^/l)	0.6 ± 1.1	0.8 ± 1.3	0.5209
HB (g/dl)	14.2 ± 1.8	14.1 ± 2.1	0.5732
HCT (%)	42.3 ± 5.2	41.6 ± 9	0.7432
MCV (fl)	84.7 ± 6.2	83.2 ± 7	0.1872
MCH (pg)	28.4 ± 2.5	27.6 ± 2.8	0.1285
MCHC (g/dl)	33.5 ± 1.2	33.1 ± 1.1	0.1832
RDW% (%)	13.2 ± 1.5	13.7 ± 1.2	0.0396*
RDWa (fl)	56.4 ± 6.8	55.7 ± 10.4	0.4991
PLT (10^9^/l)	246.9 ± 66.8	245.5 ± 63.6	0.9597
PCT (%)	0.2 ± 0.05	0.2 ± 0.05	0.7856
MPV (fl)	8.2 ± 0.8	8.6 ± 0.9	0.0377*
PDW (fl)	11.8 ± 1.2	12.3 ± 1.5	0.0602
PLCR (%)	17.9 ± 6	19.8 ± 6.6	0.0652

RBC, red blood cell; WBC, white blood cell; GRAN, granulocyte; LYM, Lymphocyte; MID, Multidimensional Inventory of Dissociation; HB, hemoglobin; HCT, hematocrit; MCV, mean corpuscular volume; MCH, mean corpuscular hemoglobin; MCHC, Mean Corpuscular Hemoglobin Concentration; RDW%, red cell distribution width; RDWa; PLT, platelet; PCT, procalcitonin; MPV, mean platelet volume; PDWs, Platelet Distribution Width; PLCR, Platelet larger cell ratio. Results are expressed as numbers or means ± SD. *Statistical significance

These results highlight the significant age and metabolic parameter differences between CAD patients and controls. Total bilirubin, ALP, glucose, troponin, and creatinine levels are higher in CAD patients, which may indicate that these substances are relevant to the pathogenesis of the disease. Conversely, the higher RDW% and MPV values in controls may reflect distinct hematological characteristics associated with the absence of CAD.

### Expression of serum MPO in CAD patients and control

Serum MPO levels were measured using ELISA; CAD patients had non-significantly lower MPO levels (*P* = 0.7291) compared to the control. Furthermore, to explore the relationship between MPO levels and disease severity within the CAD patient group, patients were stratified into three subgroups based on the severity of their coronary artery disease: single-vessel disease (SVD), double-vessel disease (DVD), and multi-vessel disease (MVD). Analysis of serum MPO levels among these groups demonstrated a non-significant difference (*P* = 0.8662), indicating that MPO levels did not exhibit significant variability based on the extent of disease involvement.

### Correlation between serum MPO levels in CAD patients and disease activity

Spearman correlation analysis revealed a statistically significant positive correlation between serum MPO levels and glucose in patients diagnosed with CAD (r = 0.2140, *P* = 0.0325). This finding suggests elevated glucose levels are associated with increased MPO levels in CAD patients. Conversely, MPO levels exhibited significant negative correlations with creatinine (r = − 0.2576, *P* = 0.0097) and total bilirubin (r = − 0.2416, *P* = 0.0155) (Table 3). These results indicate an inverse relationship between MPO levels and creatinine and total bilirubin, indicating that higher levels of these parameters are associated with lower MPO levels.

**Table 3:** Correlation between serum MPO levels and some parameters

Serum MPO level	Spearman correlation (r)	95% confidence interval	*P*-value	*P*-value summary
Age	− 0.08491	− 0.2821 to 0.1192	0.4010	ns
BMI	0.05769	− 0.1461 to 0.2568	0.5686	ns
GPT	0.009310	− 0.1931 to 0.2110	0.9268	ns
GOT	− 0.1023	− 0.2982 to 0.1019	0.3114	ns
urea	0.003468	− 0.1987 to 0.2054	0.9727	ns
Creatinine	− 0.2576	− 0.4369 to − 0.05859	0.0097	**
Total Bilirubin	− 0.2416	− 0.4230 to − 0.04153	0.0155	*
Alp	0.1817	− 0.02113 to 0.3702	0.0704	ns
LDH	0.006442	− 0.1959 to 0.2082	0.9493	ns
Glucose	0.2140	0.01250 to 0.3988	0.0325	*
Cholesterol	− 0.008797	− 0.2105 to 0.1936	0.9308	ns
TG	0.0001952	− 0.2019 to 0.2023	0.9985	ns
HDL	− 0.1250	− 0.3190 to 0.07911	0.2155	ns
LDL	− 0.07143	− 0.2696 to 0.1325	0.4800	ns
CRP	0.1777	− 0.02525 to 0.3666	0.0769	ns
CKmB	0.1223	− 0.08178 to 0.3166	0.2254	ns
Troponin	− 0.07771	− 0.2755 to 0.1263	0.4422	ns

BMI, body mass index; GPT, Glutamic Pyruvic Transaminase; GOT, glutamic-oxaloacetic transaminase; Alp, Alkaline phosphatase; LDH, Lactate dehydrogenase; TG, Triglycerides; HDL, High-density lipoprotein; LDL, Low-density lipoprotein; CRP, C-reactive protein; CKmB, creatine kinase-myoglobin binding. *Statistical significance

However, no statistically significant correlations were found between serum MPO levels and age, BMI, liver function (GPT, ALP, and GOT), renal function (urea), cellular metabolism (LDH), lipid profile (cholesterol, TG, HDL, and LDL), inflammatory marker (CRP), and cardiac biomarkers (CKmB and troponin) levels (all *P* > 0.05) (Table 3). These findings help to improve knowledge of the link between MPO and numerous clinical variables in the setting of CAD, highlighting particular factors that may or may not have a role in modifying MPO levels.

**Fig. 2:**
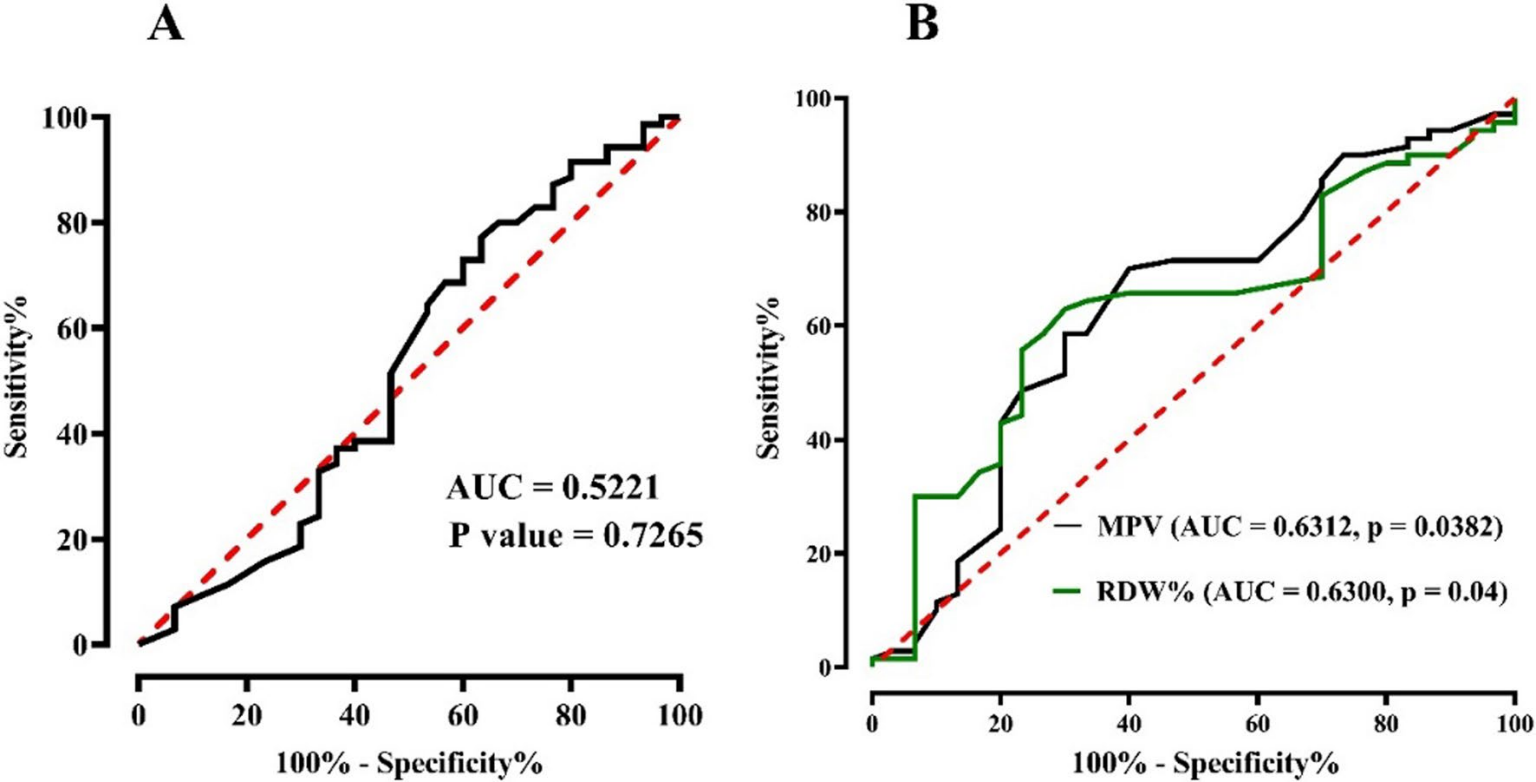
Depicts a prognostic analysis of Serum **A** MPO, **B** MPV, and RDW% levels in patients with coronary artery disease (CAD), demonstrating that serum myeloperoxidase levels exhibited a low discriminate capacity for bad prognosis based on the receiver operating characteristic curve

Moreover, this study aimed to assess the predictive value of three biomarkers (MPO, MPV, and RDW%) for coronary artery disease (CAD), as shown in Fig. 2. The findings revealed that MPO exhibited limited predictive ability, with a cut-off value of ≤ 4.84 ng/ml, an AUC of 0.5221, sensitivity of 61.43%, and specificity of 53.33% (*P* = 0.7265, DOR = 1.8201). Conversely, MPV demonstrated better predictive performance, with a significant cut-off value of ≤ 8.40, an AUC of 0.6312, a sensitivity of 70%, and a specificity of 60% (*P* = 0.0382, DOR = 3.5000). Similarly, RDW% showed promising results, with a significant cut-off value of ≤ 13.50, an AUC of 0.6300, a sensitivity of 62.86%, and a specificity of 70% (*P* = 0.0400, DOR = 3.9487).

### Genetic variations in the MPO gene: accession numbers and sequence data

Within the context of investigating genetic variations within the Myeloperoxidase (MPO) gene, a cohort of 11 individuals, comprising both controls and patients diagnosed with coronary artery disease (CAD), was meticulously studied. The specific focus was on elucidating nucleotide changes within the MPO gene and their potential association with CAD. The selected sample size of 11 individuals was a result of deliberate consideration rather than a limitation, as we aimed to explore a distinct genetic landscape with high clinical relevance. The nucleotide sequence data from the sequencing process were deposited in the National Center for Biotechnology Information (NCBI) database (BankIt). The unique accession numbers assigned to each sequence are as follows: OQ076274, OQ076275, OQ076276, OQ076277, OQ076278, OQ076279, OQ076280, OQ076281, OQ076282, OQ076283, and OQ076284. These accession numbers serve as references for the deposited genetic data, enabling easy retrieval and verification by other researchers interested in our study. The data are available to the scientific community for further analysis and replication.

**Fig. 3:**
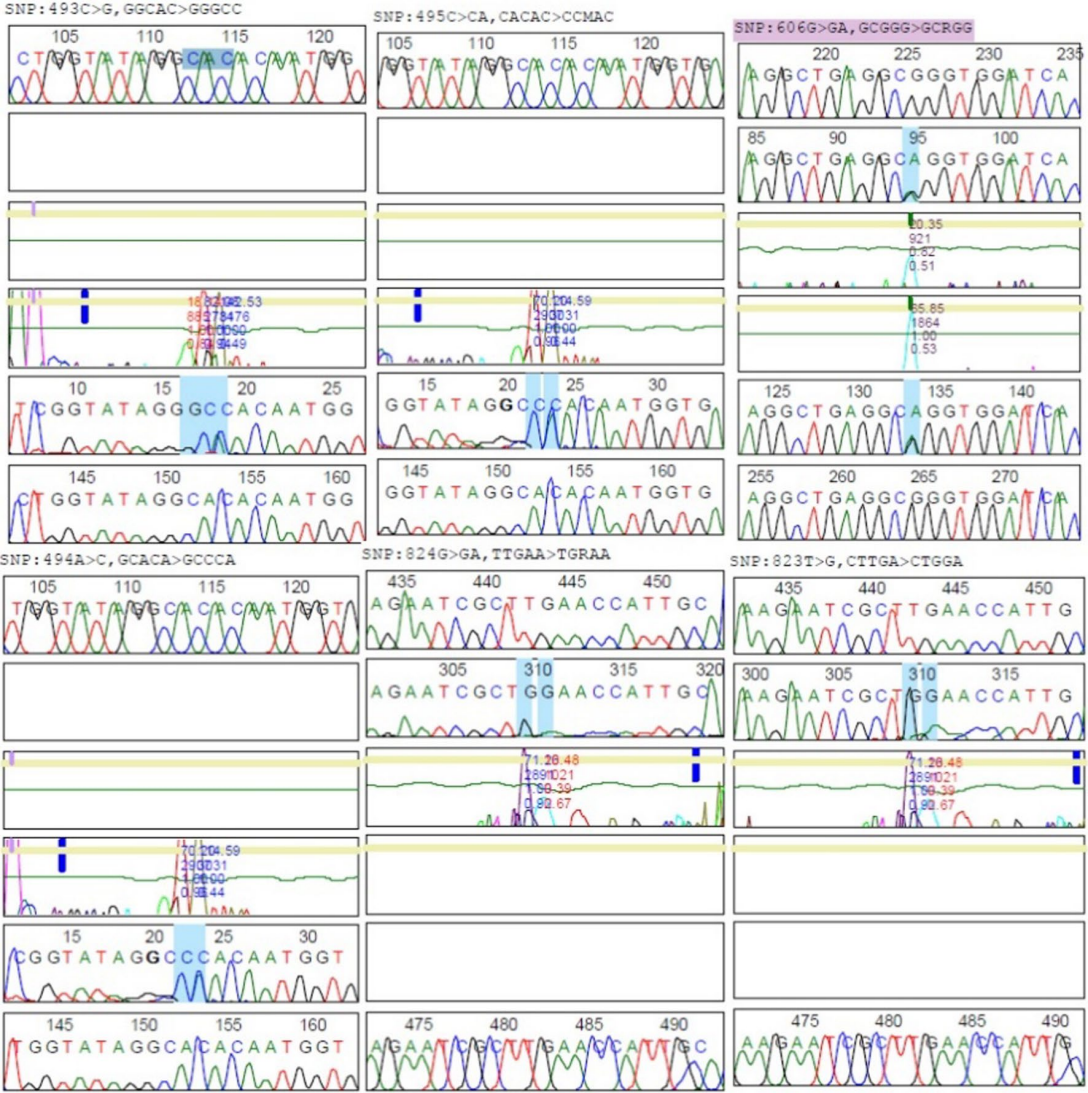
Summary images of MPO Sequenced PCR products SNPs rs2333227 polymorphism. Showing reference numbers in the first row and patient sequences in the other rows analyzed with Mutation Surveyor software. Consecutive peaks correspond to DNA fragments differing by one base, and each line corresponds to one given nucleotide. Automated data analysis allows the determination of the sequence (symbols above the peaks)

Upon analyzing the sequences, recording the genotypes as “GA, GG, GA, GG, GA, GG, AA, GA, GG, GG, and GA,” respectively. The wild-type genotype is denoted as GG, while variations involve a G to A change at specific positions in the MPO gene. Among the 11 samples, five individuals exhibited the wild-type genotype (GG), indicating the absence of identified variations. Conversely, six individuals displayed variations where G was changed to A at specific positions. Notably, one sample showed both genotypes changed to A, suggesting the presence of multiple variations in that particular individual. Figure 3 illustrates the six transcript variants resulting from single nucleotide changes at specific positions in the MPO gene: 493C > G, 494A > C, 495C > CA, 606G > GA, 823T > G, and 824G > GA. These single nucleotide changes underlie the observed genetic variations within the MPO gene. Comprehensive sequencing data analysis was performed using Mutation Surveyor V5.1.2 software, a widely-used tool in genetic research. This software facilitated the detecting and characterizing of genetic variations in the sequenced MPO gene data. The deposited nucleotide sequence data, represented by the assigned accession numbers, are available for public access in the NCBI database, ensuring transparency and promoting further research in this field.

## Discussion

The current study contributes significant findings regarding patients with angiographically confirmed CAD compared to the control group. Firstly, consistent with previous research, CAD patients were observed to be notably older and predominantly male, emphasizing the predictive value of age, sex, and symptoms in CAD diagnosis [13, 14]. This aligns with the established understanding that advanced age and male gender are associated with a higher risk of developing CAD.

Furthermore, an analysis of clinical parameters in this study indicated significant elevations in serum Glucose, Total bilirubin, alp, creatinine, and troponin levels among the CAD group compared to the controls. These results support the findings of Doganer et al. and Liang et al., who suggested that liver function tests could serve as sensitive indicators for assessing the burden of atherosclerosis [15, 16]. By highlighting these alterations in clinical parameters, the study enhances our understanding of the specific physiological changes associated with CAD.

Also, the study revealed that CAD patients exhibited significantly lower levels of RDW% and MPV. This finding aligns with the lower MPV levels reported by Wada et al. [17]. However, it is worth noting that the study conducted by Temelli et al. did not find a statistically significant difference in RDW% levels between the study groups [18]. These findings suggest that RDW% and MPV may serve as potential markers for CAD. Moreover, these findings contribute to the existing body of knowledge by reaffirming the association between CAD and advanced age, male gender, and specific alterations in clinical parameters. Furthermore, they support the notion that liver function tests may offer valuable insights into the presence and severity of atherosclerosis. Conversely, the higher RDW% and MPV values in controls may reflect distinct hematological characteristics associated with the absence of CAD.

While systemic MPO levels have been associated with poor prognosis in acute CAD patients, studies have shown that elevated systemic MPO levels are linked to increased cardiovascular events, such as myocardial infarction, heart failure, and mortality, in patients with acute CAD [10, 19–21]. However, this study's findings revealed no significant elevation of serum MPO levels in CAD patients compared to the control group. Similar results have been reported in other studies with larger patient cohorts [22–24]. It is possible that the detection of circulating MPO underestimates the actual amount of MPO sequestered within the vessel wall, as MPO has been shown to rapidly bind to heparan-sulfated glycosaminoglycans (GAGs) and deposit within the sub endothelium of the vessel wall, suggesting increased sequestration of leukocyte-derived MPO in CAD patients [25, 26]. This may explain why CAD patients exhibit lower MPO levels in polymorphonuclear (PMN) cells than controls in acute and stable CAD cases [9, 27, 28]. Moreover, aspirin, a commonly prescribed medication for individuals with coronary artery disease (CAD), may help explain decreased myeloperoxidase (MPO) levels in CAD patients. Aspirin is an anti-platelet medication and non-steroidal anti-inflammatory drug (NSAID) frequently used to prevent acute coronary syndrome (ACS) and reduce mortality in CAD patients [29]. Its mechanism of action involves inhibiting prostaglandin H synthase, an enzyme responsible for synthesizing prostaglandins and thromboxane from arachidonic acid, through the actions of cyclooxygenase and peroxidase [30, 31]. During the study, it was noted that 72.9% of CAD patients interviewed were taking aspirin as part of their medication regimen. Additionally, another study revealed that aspirin use was associated with decreased neutrophil/lymphocyte ratio and reduced levels of pMPO (a marker of MPO activity). These findings offer further insights into the potential benefits of aspirin in preventing myocardial infarction (MI) and reducing mortality [30]. Previous research has also demonstrated aspirin's ability to decrease MPO activity and neutrophil levels [32, 33].

The correlation analysis demonstrated a noteworthy negative association between MPO and both creatinine and Total Bilirubin. Notably Yin, et al. [34] reported a positive relationship between total bilirubin and major adverse cardiovascular events. Additionally, our findings revealed a negative correlation between creatinine and MPO, in line with previous research [35]. Conversely, a significant positive correlation was observed between MPO and Glucose, consistent with similar results reported by Qaddoumi et al. [36].

Moreover, the novel findings of this study highlight the potential of mean platelet volume (MPV) and red cell distribution width (RDW%) as predictive markers for severe atherosclerosis and as tools for identifying cardiac risks in CAD patients. While direct comparisons with existing data are challenging due to the absence of available comparative studies in the literature, these results emphasize the need for further research to explore the diagnostic significance of MPV and RDW% in CAD patients.

Several reports have shown that RDW levels are related to cardiovascular diseases [37]. Red cell distribution width reflects variability in the size of circulating erythrocytes. New young RBC production during anemia results in increased RDW. However, besides anemia, RDW may also be affected by other conditions including inflammatory diseases, iron deficiency, hemolysis and renal failure [37] and also cardiovascular diseases [38, 39]. On the hand, in patients with normal platelet counts, a high MPV is suggestive of primary thrombocytosis, while a low MPV characterizes a reactive thrombocytosis, seen in infection, inflammation, or malignancy [40].

Finally, identifying gene variants in the MPO gene expands our understanding of the genetic diversity within this gene and its potential implications for CAD susceptibility. The necessity of further examining the functional implications of MPO gene variations in CAD pathogenesis is highlighted by previous research that have connected MPO gene variations with higher risk for CAD [41], and other studies that have found no association between MPO polymorphism and CAD severity [24]. Genetic polymorphisms and MPO gene enzymatic activity have been linked to numerous diseases and biological processes [42]. Mäkelä et al.’s investigation showed that MPO gene polymorphisms result in genotypes with high (G/G) and low-expression (A/A, A/G) levels [41]. Additionally, those who had the G allele had lower levels of atherosclerosis risk factors than those who carried the A allele [41]. These findings highlight the possible impact of MPO genetic variants on parameters associated to atherosclerosis and disease risk. Understanding the impact of MPO gene polymorphisms on disease development and risk factors provides valuable insights for personalized treatment and preventive strategies in affected individuals.

## Conclusions

In conclusion, this study provides valuable insights into the association between MPO levels and CAD, incorporating clinical parameters and genetic analysis. However, it is essential to acknowledge the limitations of the study, including the relatively small sample size. Future research endeavors should prioritize larger cohorts and employ functional analyses to validate our findings. Furthermore, the study lays the groundwork for understanding the potential diagnostic and prognostic value of Red Cell Distribution Width (RDW%), Mean Platelet Volume (MPV), and MPO levels in CAD. While these results are promising, further investigations are warranted to comprehensively validate the utility of these biomarkers in clinical settings. Additionally, exploring the role of MPO gene variants in CAD onset and progression merits attention in future research endeavors. This study serves as a stepping stone, and we emphasize the importance of ongoing efforts to advance our understanding of these biomarkers, ultimately contributing to improved diagnostic and therapeutic strategies for CAD.

## Data Availability

The nucleotide sequence data from the sequencing process were deposited in the National Center for Biotechnology Information (NCBI) database (BankIt). The unique accession numbers assigned to each sequence are as follows: OQ076274, OQ076275, OQ076276, OQ076277, OQ076278, OQ076279, OQ076280, OQ076281, OQ076282, OQ076283, and OQ076284.

## Abbreviations

CAD: Coronary artery disease
MPO: Myeloperoxidase
ELISA: Enzyme-linked immunosorbent assay
RDW%: Red cell distribution width
MPV: Mean platelet volume
WHO: World Health Organization
CVDs: Cardiovascular diseases
SNP: Single nucleotide polymorphism
PCR: Polymerase chain reaction
EDTA: Ethylenediaminetetraacetic acid
ALT: Alanine aminotransferase
AST: Aspartate aminotransferase
ALP: Alkaline phosphatase
LDH: Lactate dehydrogenase
BMI: Body mass index
TG: Triglycerides
HDL: High-density lipoprotein
LDL: Low-density lipoprotein
CRP: C-reactive protein
CK-MB: Creatine kinase-MB
RNA: Ribonucleic acid
DNA: Deoxyribonucleic acid
PCR: Polymerase chain reaction
UV: Ultraviolet
NCBI: National Center for Biotechnology Information
GAGs: Glycosaminoglycans
PMN: Polymorphonuclear
NSAID: Non-steroidal anti-inflammatory drug
ACS: Acute coronary syndrome
MI: Myocardial infarction
